# Supplementary material for: Giant uniaxial negative thermal expansion in FeZr2 alloy over a wide temperature range
Source: Nat Commun. 2023 Jul 24;14:4439. doi: 10.1038/s41467-023-40074-7 (PMC10366141; doi:10.1038/s41467-023-40074-7)
Supplement: Supplementary file 1 — Supplementary Information [file 41467_2023_40074_MOESM1_ESM.pdf]

Supporting Information for

Giant uniaxial negative thermal expansion in $FeZr_2$ alloy over a wide temperature range

Meng Xu[1], Qiang Li[2], Yuzhu Song[1], Yuanji Xu[3], Andrea Sanson[4,5], Naike Shi[1], Na Wang[1], Qiang Sun[6], Changtian Wang[1], Xin Chen[2], Yongqiang Qiao[6], Feixiang Long[1], Hui Liu[1], Qiang Zhang[7], Alessandro Venier[4], Yang Ren[8], Francesco d'Acapito[9], Luca Olivi[10], Danilo Oliveira De Souza[10], Xianran Xing[2], Jun Chen[1,11*]

[1] Beijing Advanced Innovation Center for Materials Genome Engineering, Department of Physical Chemistry, University of Science and Technology Beijing, Beijing 100083, China

[2] Institute of Solid State Chemistry, University of Science and Technology Beijing, Beijing 100083, China

[3] Institute for Applied Physics, University of Science and Technology Beijing, Beijing 100083, China

[4] Department of Physics and Astronomy, University of Padua, Padova I-35131, Italy

[5] Department of Management and Engineering, University of Padua, Padova I-35131, Italy

[6] International Laboratory for Quantum Functional Materials of Henan, School of Physics and Engineering, Zheng-zhou University, Zhengzhou 450001, China

[7] Neutron Scattering Division, Oak Ridge National Laboratory, Oak Ridge, TN, 37831, USA

[8] Department of Physics, City University of Hong Kong, Kowloon, Hong Kong, Hong Kong, 518057, China

[9] CNR-IOM-OGG c/o European Synchrotron Radiation Facility (ESRF) 71 Av. des Martyrs, 38000 Grenoble, France.

[10] ELETTRA Synchrotron Trieste, s.s. 14 km 163,500 in Area Science Park, 34149 Basovizza - Trieste, Italy

[11] Hainan University, Haikou 570228, Hainan Province, China

*Corresponding author: junchen@ustb.edu.cn

Contents

1. Thermal expansion behavior and detailed structural information for the MZr_2 (M = Fe, Ni) systems.

1.1 The linear thermal expansion of MZr_2 (Figs. S1-S2)

1.2 The SXRD, NPD results of MZr_2, and nPDF for $FeZr_2$ (Figs. S3-S5)

1.3 The structure evolution of temperature dependence of $FeZr_2$ and $NiZr_2$ (Figs. S6-S8, Table S1)

2. The morphology and grain orientation of $FeZr_2$ ingot (Fig. S9)

3. Results of phonon

3.1 The ADPs and phonon dispersion of results of MZr_2 (Figs. S10-S12)

4. EXAFS results and analysis

4.1 Temperature dependence XANES for different elements for MZr_2 systems (Fig. S13)

4.2 The fitting simulation for different elements and parameters of MZr_2 (Fig. S14-S17, Tables S2-S6)

5. First-principles calculations

5.1 Charge distribution of $FeZr_2$ (Fig. S18)

5.2 The energy bands, DOS, and -COHP of MZr_2 systems (Figs. S19-S21, Tables S7-S8)

1. Thermal expansion behavior and detailed structural information for the MZr_2 (M = Fe, Ni) systems

In order to provide insights into the mechanism of $FeZr_2$ with giant uniaxial (1D) NTE over a wide temperature range, here, we comparatively studied the isostructural MZr_2 systems. It can be found that there is a vastly different in the anisotropic thermal expansion between the two materials (Fig. S2 and Fig. S6).

1.1 The linear thermal expansion of MZr_2

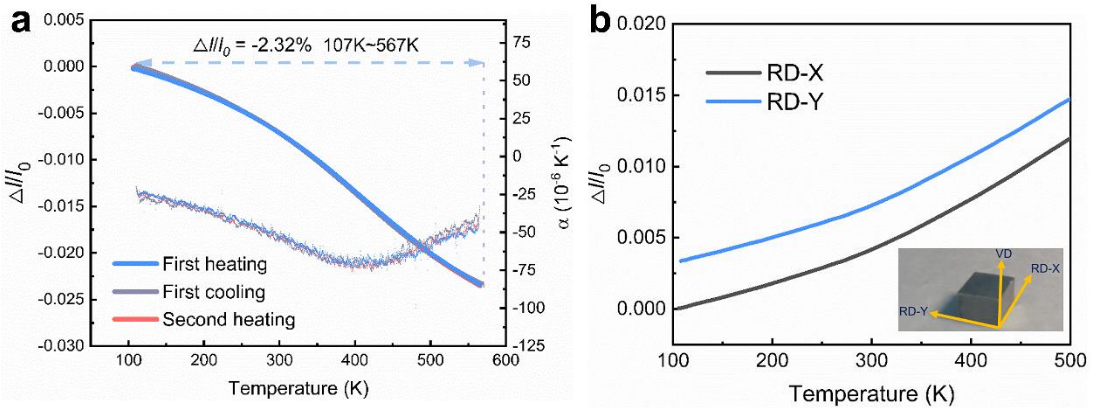

Fig. S1. The linear thermal expansion measurements of $FeZr_2$ ingot. (a) Thermal cycling measurement represented by the different colors of solid lines, and the corresponding coefficient of thermal expansion (CTE) represented by the dot. The sample is first heated, then cooled, and finally heated in a cycle. (b) The linear thermal expansion of $FeZr_2$ ingot along mutually perpendicular directions (denoted RD-X and RD-Y, respectively) inside the RD-RD plane. The inset shows the sample diagram and the reference coordinate system.

Fig. S1a shows that $FeZr_2$ ingot has hysteresis-free thermal cycling performance between 107 K to 567 K. Fig. S1b shows the linear thermal expansion within the RD-RD plane for $FeZr_2$ determined by dilatometry along mutually perpendicular directions (RD-X and RD-Y). The ingot exhibits strong positive thermal expansion (PTE) with the CTE of $\bar{\alpha}_{l\text{-}X} = 30.59 \pm 0.02 \times 10^{-6}$ K^{-1} and $\bar{\alpha}_{l\text{-}Y} = 29.26 \pm 0.02 \times 10^{-6}$ K^{-1} in the RD-X and RD-Y directions between 107 to 500K, respectively. As a contrast, the average CTE for lattice parameter *a* is $\alpha_a = 25.75 \pm 0.04 \times 10^{-6}K^{-1}$ for $FeZr_2$ between 10 to 500K ($\alpha_a = 29.75 \pm 0.04 \times 10^{-6}K^{-1}$, 100K-500K) extracted from the NPD (Fig. S6a). These

result in the planar thermal expansion of the ingot showing nearly the same CTE as the *a*-axis. Since $FeZr_2$ is tetragonal crystal symmetry. And the EBSD indicates that its grain orientations of [001] and [110] are random distribution inside the RD-RD plane.

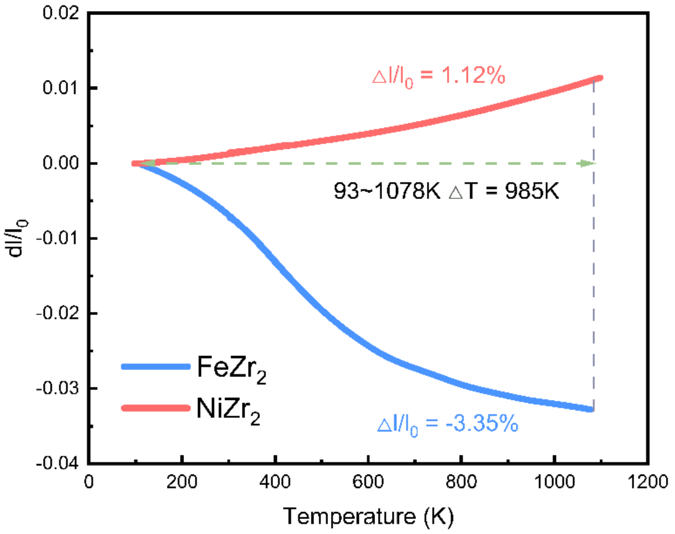

Fig. S2. Contrast the thermal expansion of the ingot $FeZr_2$ and $NiZr_2$ systems along the VD.

As previously reported, the sample cooling process will produce a strong texture due to large temperature gradients.[1] For example, a giant anisotropic magnetocaloric effect can be achieved by the arc melting method.[1] The targeted sample $FeZr_2$ was prepared by arc melting in the copper crucibles under a high-purity argon atmosphere. The upper surface is heated by the electric arc. And the bottom of the sample is in contact with the copper crucible, which results in an extreme temperature difference between the top and bottom surfaces of the sample. It will produce a vast temperature gradient between the upper and bottom surfaces of the sample. This results in $FeZr_2$ ingots with a strong texture.

1.2 The SXRD, NPD results of MZr_2, and nPDF for $FeZr_2$

The SXRD patterns of MZr_2 measured at room temperature (RT) could refine well using a single *I*4/*mcm* structure by the Rietveld refinement method (Fig. S3), indicating that both components are pure phase and possess the same crystal structure. Except for the crystal structure diffraction peak, no new magnetic or diffraction peak mutation was

found at the NPD measurement for all components (Fig. S3 and S8), indicating no magnetism for $MrZr_2$ systems. The conclusion of no magnetism is consistent with the previous study about the MZr_2 systems.[2,3]

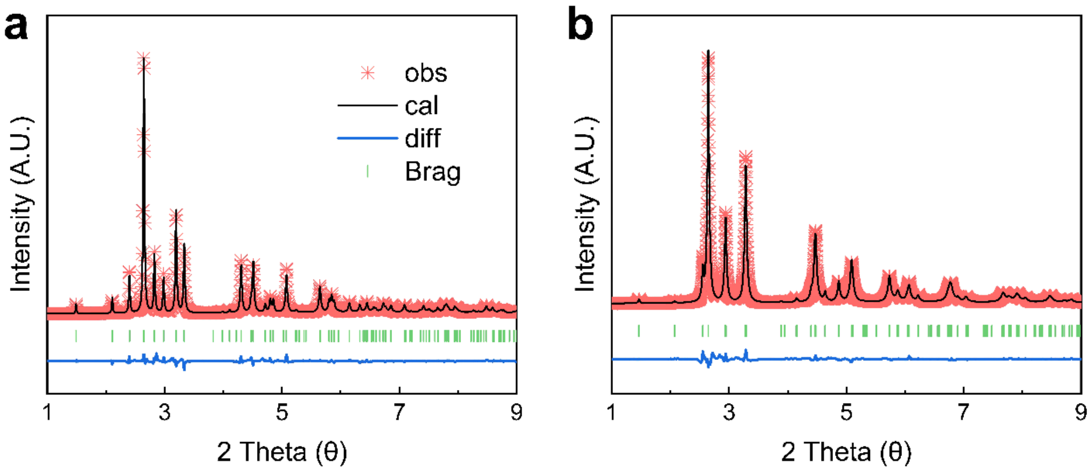

Fig. S3. The Rietveld refinements of SXRD patterns using a tetragonal model at room temperature (RT) for (a) $FeZr_2$ and (b) $NiZr_2$.

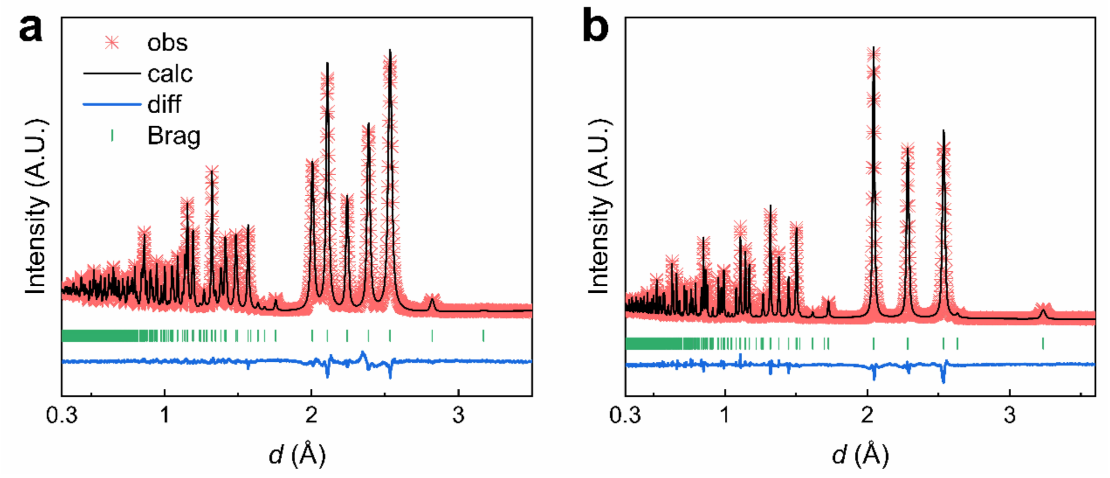

Fig. S4. The Rietveld refinements of NPD patterns at 10K using the tetragonal model for (a) $FeZr_2$ and (b) $NiZr_2$.

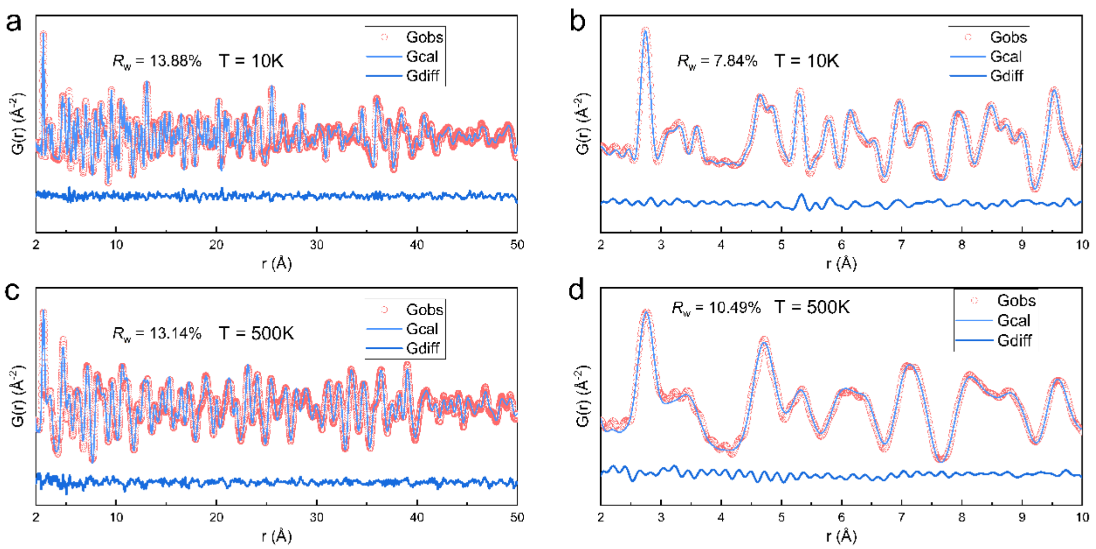

Fig. S5. Pair distribution function (PDF) fit of neutron total scattering for a large r range between 2 to 50 Å for $FeZr_2$ obtained at (a) 10K and (c) 500K. A short r range between 2 to 10 Å at (b) 10K and (d) 500K. The tetragonal (*I*4/*mcm*) model was used for the different temperatures and fitting ranges. The pink circles and blue lines represent the observed and calculated values, respectively. The bottom of the figures shows the difference between the experimental and calculated value.

Both different ranges of r at a large interval (2~50 Å) or short interval (2~10 Å) at different temperatures can be well fitted with the same structure (*I*4/*mcm*) model by pdfgui (Fig. S5), indicating the average and local structures of $FeZr_2$ are consistent.

Furthermore, the structure of $FeZr_2$ has good structural stability in the measurement temperature range.

1.3 The structure evolution of temperature dependence of $FeZr_2$ and $NiZr_2$.

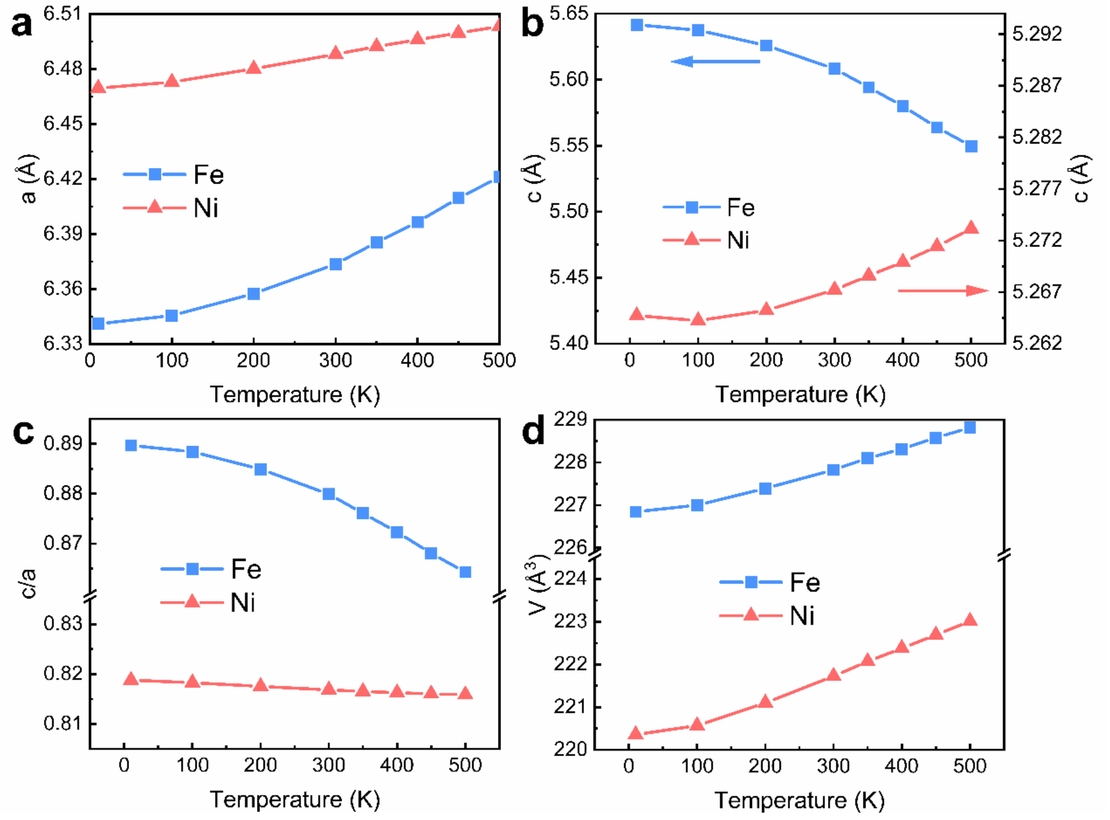

Fig. S6. Temperature dependence of lattice parameters for $FeZr_2$ and $NiZr_2$ extracted from NPD. (a) Temperature dependence of lattice constant of *a*, (b) lattice constant of *c*, (c) the axial ratio of *c*/*a*, and (d) unit cell volume.

The average CTEs for lattice parameter *a* are $\alpha_{Fe,a}$ = 25.75 ± 0.04 × $10^{-6}K^{-1}$ and $\alpha_{Ni,a}$ = 10.60 ± 0.03 × $10^{-6}K^{-1}$, for lattice parameter *c* are $\alpha_{Fe,c}$ = -33.47 ± 0.05 × $10^{-6}K^{-1}$ and $\alpha_{Ni,c}$ = 3.27 ± 0.04 × $10^{-6}K^{-1}$ for $FeZr_2$ and $NiZr_2$ between 10 to 500K, as shown in Fig. S6a-b, respectively. On the other hand, in comparison of variable temperature lattice parameters of MZr_2 (M = Fe, Ni) systems, with increasing M atomic number, a giant NTE converts to normal PTE along the *c*-axis from $FeZr_2$ to $NiZr_2$ (Fig. S6b). Noteworthy, there is a temperature region of NTE below 100K in the *c*-axis of $NiZr_2$, suggesting the factors triggering NTE in $FeZr_2$ as well as contribute weakly to $NiZr_2$. Corresponding to the *c*-axis NTE, the *a*-axis transitions from a large PTE to a normal

PTE (Fig. S6a). Fig. S6c shows the variable temperature axial ratios (*c*/*a*), discovering that $FeZr_2$ has a large *c*/*a* than $NiZr_2$. Interestingly, the difference in the atomic radius of Fe compared to that of Ni is negligible[4]. Still, the lattice parameter *c* of $FeZr_2$ is much larger than $NiZr_2$ at room temperature (Table S1). It indicates, with increasing temperature, the rapidly decreasing *c*/*a* of $FeZr_2$ indicates that a large *c*/*a* is necessary to produce a large 1D NTE.

Moreover, the two materials exhibit vastly different anisotropic thermal expansion, but the intrinsic volumetric thermal expansion of $FeZr_2$ and $NiZr_2$ is a little different (Fig. S6d), and the α_V is $17.73 \pm 0.07 \times 10^{-6}$ K^{-1} and $24.60 \pm 0.04 \times 10^{-6}$ K^{-1} between 10K to 500K corresponding to $FeZr_2$ and $NiZr_2$, respectively.

Table S1. The Rietveld refinement results of MZr_2 (M = Fe, Ni) systems at room temperature (RT) were obtained by NPD.

	Space group	*a* (Å)	*c* (Å)	*c*/*a*	M-M (Å)
$FeZr_2$	*I*4/*mcm*	6.37363	5.60829	0.879921	2.804145
$NiZr_2$		6.48812	5.26723	0.811827	2.633615

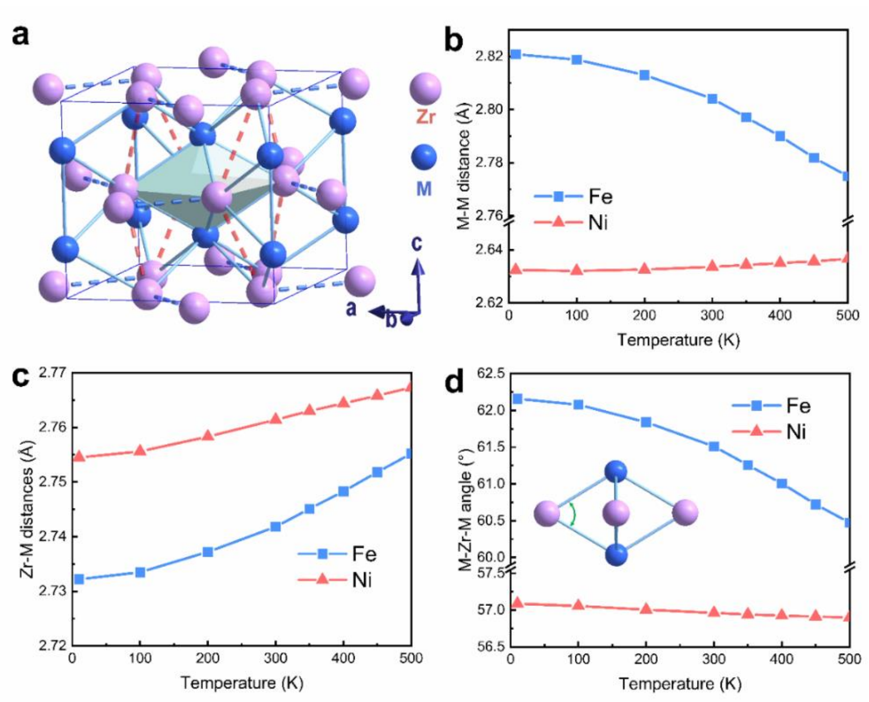

Fig. S7. Analysis of the temperature dependence of crystal structure of MZr_2 (M = Fe, Ni) obtained from NPD. (a) The crystal structure of MZr_2. The temperature dependence of (b) the M-M distances, (c) the Zr-M distances, and (d) the M-Zr-M angle.

The crystal lattice parameters (*a*, *c*, and V) for $FeZr_2$ and $NiZr_2$ are dominated by the M_2Zr_4 octahedral. In the M_2Zr_4 octahedral, the Zr-Fe bond has a larger PTE than the Zr-Ni bond (Fig. S7c), but the Fe-Zr-Fe angle decreases faster (Fig. S7d). This means that the Fe_2Zr_4 octahedral exhibits faster compression. For the *ab*-plane thermal expansion, it can be found that the thermal expansion of the Zr-M bond in the M_2Zr_4 octahedral decides the PTE magnitude in the *ab*-plane. Besides, it can be found that the Ni-Ni bond is a normal magnitude PTE, while the Fe-Fe bond has a strong NTE (Fig. S7b). Since the length of the M-M bond is half of the lattice parameter of *c*, the change in thermal expansion of the M-M bond determines the thermal expansion magnitude of the *c*-axis. In summary, the lattice thermal expansion behavior of the MZr_2 can be determined by the primary unit M_2Zr_4 octahedral.

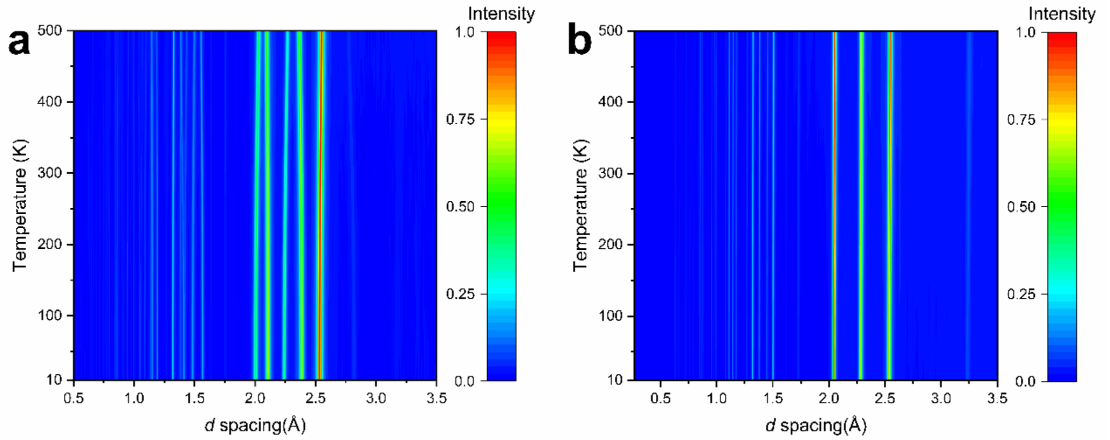

Fig. S8. Contour plot of the normalized NPD patterns (10K~500K) for the targeted samples (a) $FeZr_2$ and (b) $NiZr_2$.

The temperature dependence of NPD patterns (10K~500K) for $FeZr_2$ and $NiZr_2$ are shown in Fig. S8. It can be found the intensity of the diffraction peaks changes negligibly, and there are no excess diffraction peaks appear except for the lattice diffraction peaks, which indicates that both samples are no magnetic properties in the

measurement temperature region. Besides, it is found only the displacement of the diffraction peak without the splitting of the diffraction peak under this measured temperature region. These results can exclude the magnetovolume effect and phase transitions that produce NTE in MZr_2 (M = Fe, Ni) systems.

2. The morphology and grain orientation of MZr_2 ingot

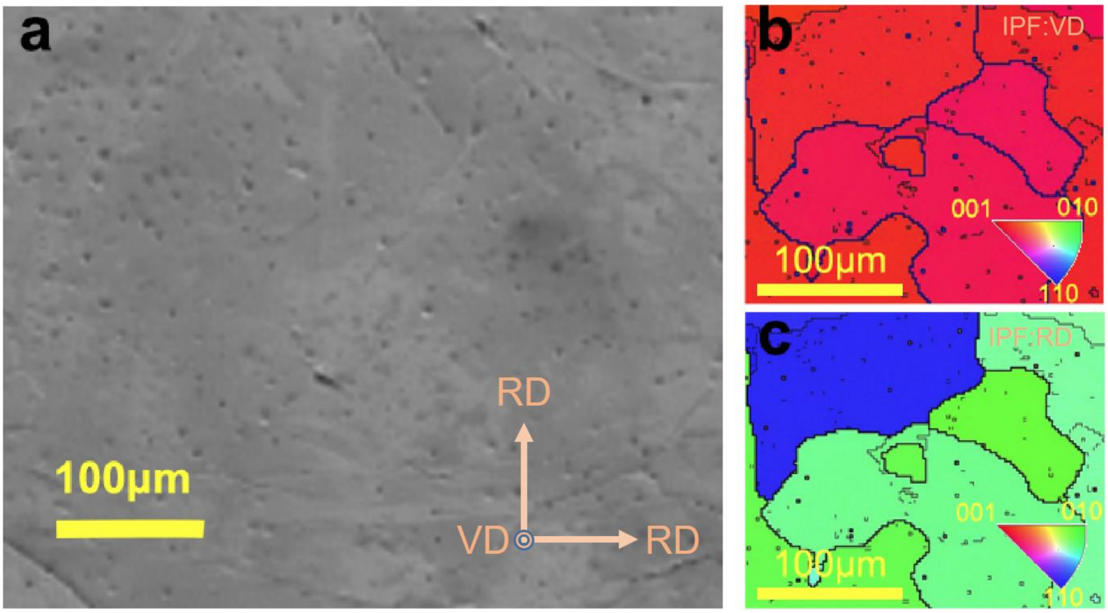

Fig. S9. EBSD measurement of $FeZr_2$. (a) The SEM using the secondary electron emission model for $FeZr_2$ inside the RD-RD plane. (b-c) The IPF of electron back-scattering diffraction (EBSD) patterns along the vertical direction (VD) and the radial direction (RD) of the $FeZr_2$ ingot. The EBSD samples were prepared using the electropolishing method, which exists in small pits due to a long corrosion time.

The morphology of $FeZr_2$ alloy was examined using SEM with the secondary electron emission model inside the RD-RD plane (Fig. S9a). It can be found that there was no discernible boundary at the grain boundary, indicating that no other phase precipitated at the grain boundary. In the corresponding region of SEM measurement, the EBSD measurements at RD-RD (Figs. S9b-c) plane reveal a strong texture in $FeZr_2$ grains with the orientation of [001]//VD, and the grain orientations of [010] and [110] are randomly distributed in the RD-RD plane.

3. The ADPs and phonon dispersion of results of MZr_2

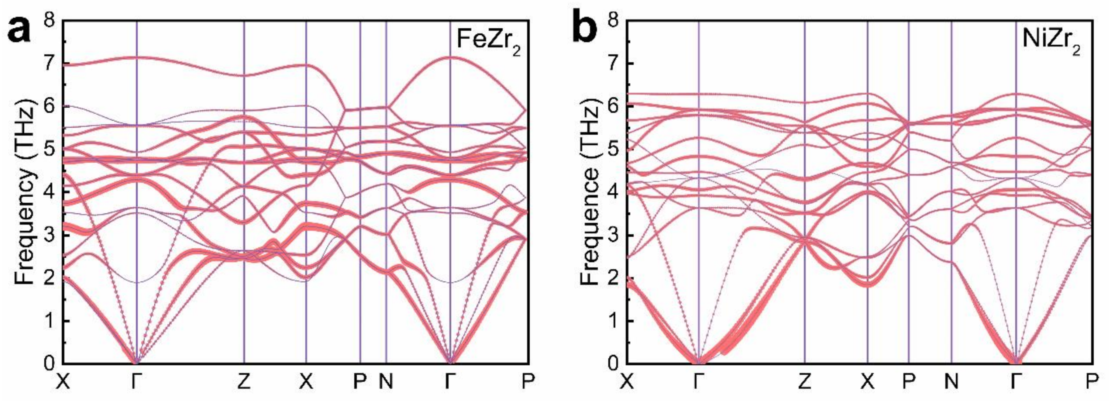

Fig. S10. The phonon dispersion curves for $FeZr_2$ (**a**) and $NiZr_2$ (**b**), with the size of the dots corresponding to the magnitude of the Grüneisen parameters along the *a*-axis (γ_a), the red color indicates positive γ_a and the blue color indicates negative γ_a.

Al the γ_a are positive for both $FeZr_2$ and $NiZr_2$ (Fig. S10), indicating that driven by phonons in the *ab*-plane is the PTE. This is consistent with the thermal expansion along the *a*(*b*)-axis measured by the experiments.

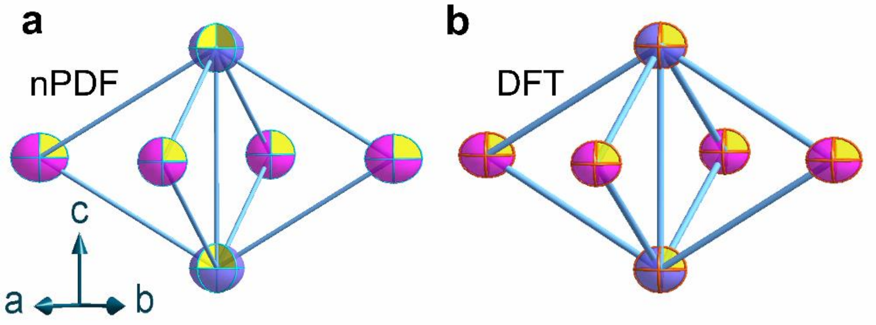

Fig. S11. Thermal ellipsoids for $FeZr_2$ acquired by the nPDF (a) and phonon dispersion simulations (b) at 300K. The experiments and calculations show that the thermal vibrational ellipsoids of Zr and Fe are almost identical.

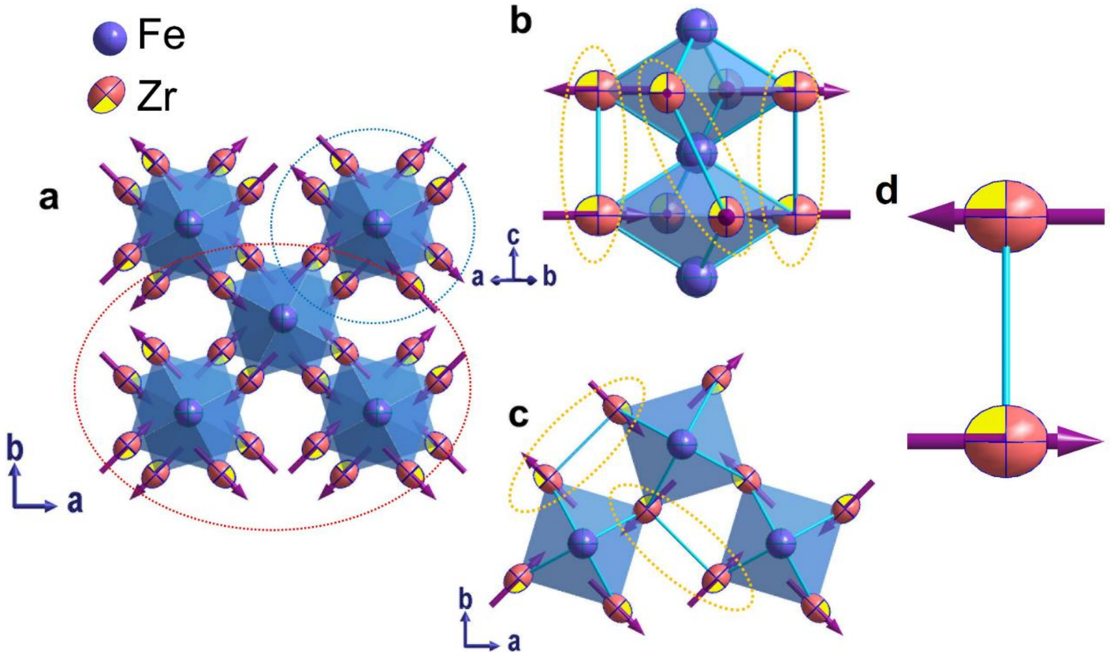

Fig. S12. Schematic of the atomic vibrations of $FeZr_2$. (a) The atomic displacement parameters (ADPs) schematic of Zr and Fe in $FeZr_2$ from nPDF at 300K. The ellipsoid size represents the amplitude. The directions of the arrow are the vibration mode of $FeZr_2$ at 63 cm^{-1}. (b,c) The partial enlargement schematics correspond to the blue (b) and red (c) line areas. The thin cyan rods in the structure represent chemical bonds. (d) The ellipsoids schematic of the Zr atom in one Zr-Zr bond, which denotes the yellow dashed line areas in (b) and (c). The arrow represents the direction of vibration.

It can be found that the vibration directions of Zr atoms in $FeZr_2$ prefer transverse vibrations for the Zr-Zr bonds (Fig. 12b,c,d). It is due to the Zr-Zr bonds (distances less than 3.2Å) having relatively strong interactions (Table S7), which make the Zr-Zr bonds more inclined to bend rather than stretch.

4. EXAFS results and analysis

4.1 Temperature dependence XANES for different elements for MZr_2 systems

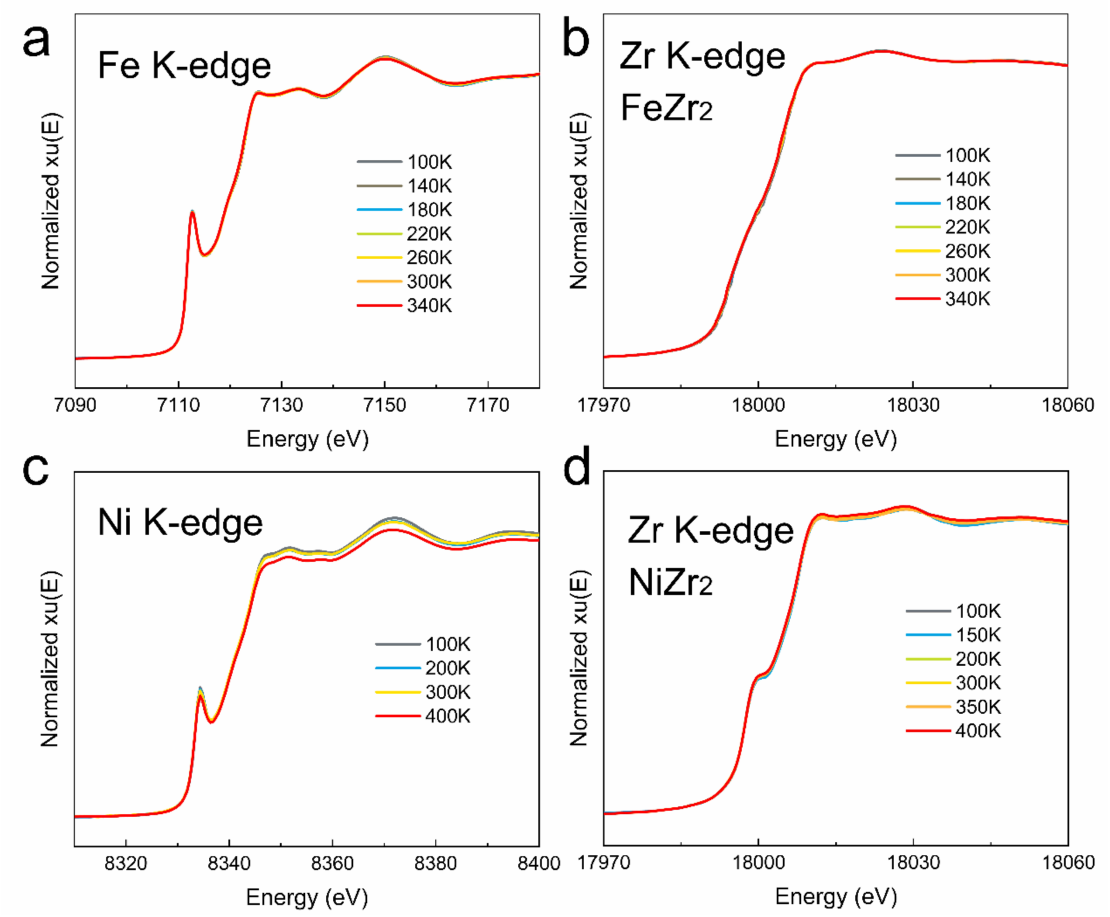

Fig. S13. Temperature dependence of K-edge XANES spectra for (a) Fe and (b) Zr of $FeZr_2$, (c) Ni and (d) Zr of $NiZr_2$.

It can be found that there are no changes in the variable temperature K-edge XANES spectra change for all the elements (Fig. S13), indicating that 1D NTE due to electron transfer is excluded.

4.2 The fitting simulation for different elements and parameters of MZr_2

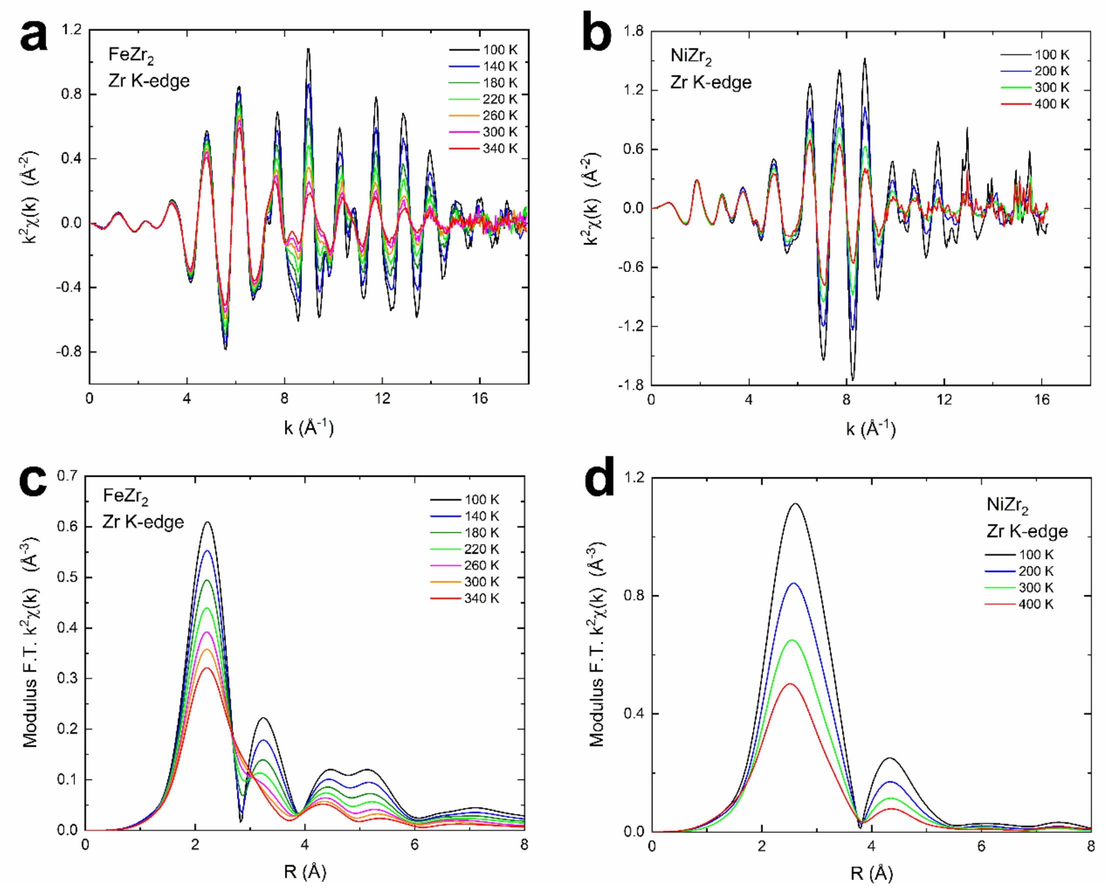

Fig. S14. Zr K-edge EXAFS data analysis for MZr_2 (M = Fe, Ni) systems. The Zr K-edge EXAFS signals of **(a)** $FeZr_2$ and **(b)** $NiZr_2$. The Fourier Transform of Zr K-edge EXAFS data for **(c)** $FeZr_2$ and **(d)** $NiZr_2$. It was performed in the k-range 2.5-12 $Å^{-1}$ with k^2-weight and Gaussian window.

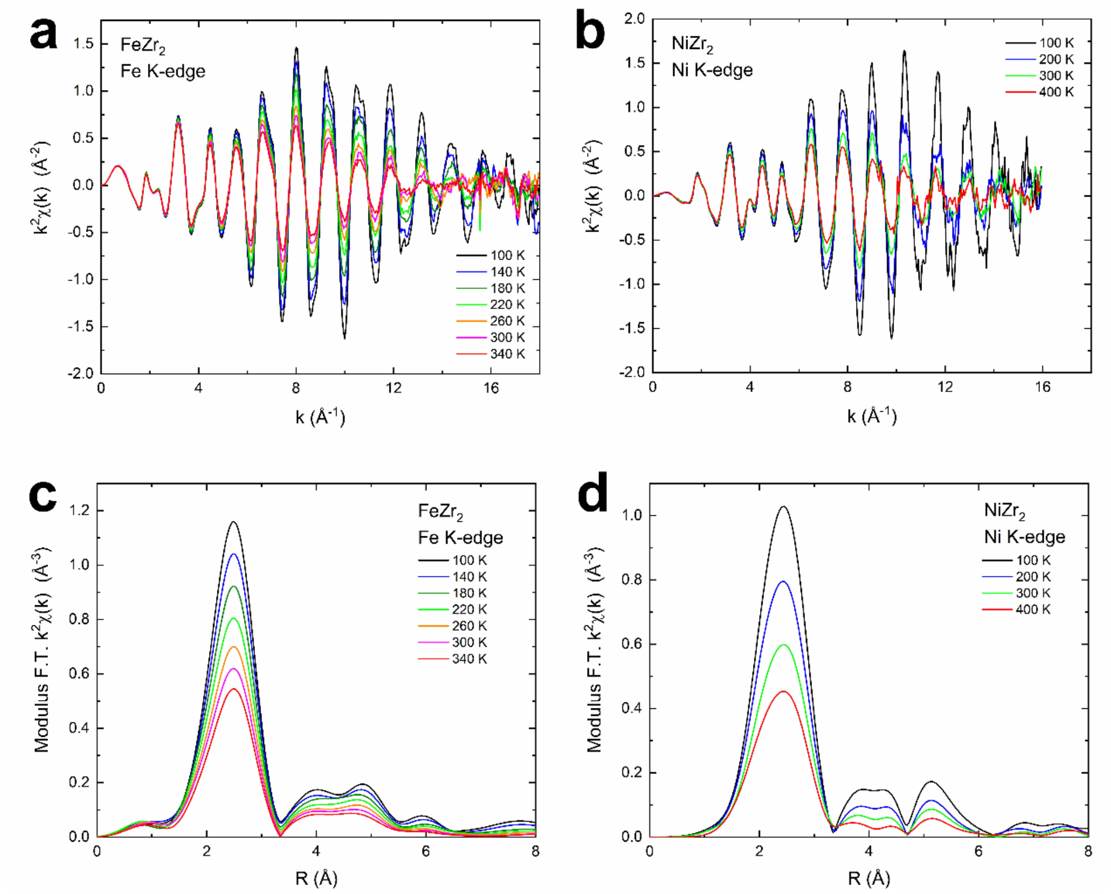

Fig. S15. Fe and Ni K-edge EXAFS data analysis for MZr_2 (M = Fe, Ni) systems. **(a)** Fe and **(b)** Ni K-edge EXAFS signals. The Fourier Transform of **(c)** Fe and **(d)** Ni K-edge EXAFS signals. It was performed in the k-range 2.5-12 $Å^{-1}$ with k^2-weight and Gaussian window.

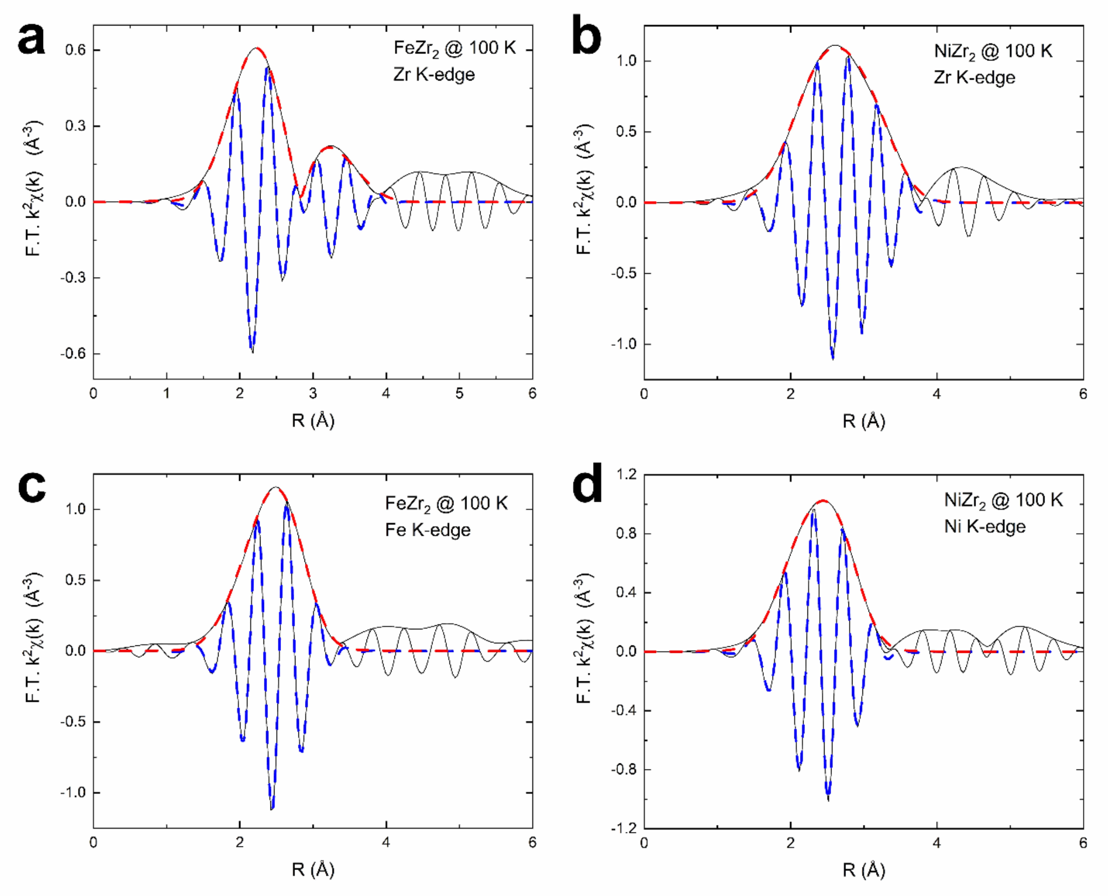

Fig. S16. Example of best-fitting simulations for EXAFS data at 100K. Zr K-edge of **(a)** $FeZr_2$ and **(b)** $NiZr_2$. **(c)** Fe and **(d)** Ni K-edge. EXAFS signals are continuous lines. Best-fitting simulations of the peaks are the dashed-bold lines.

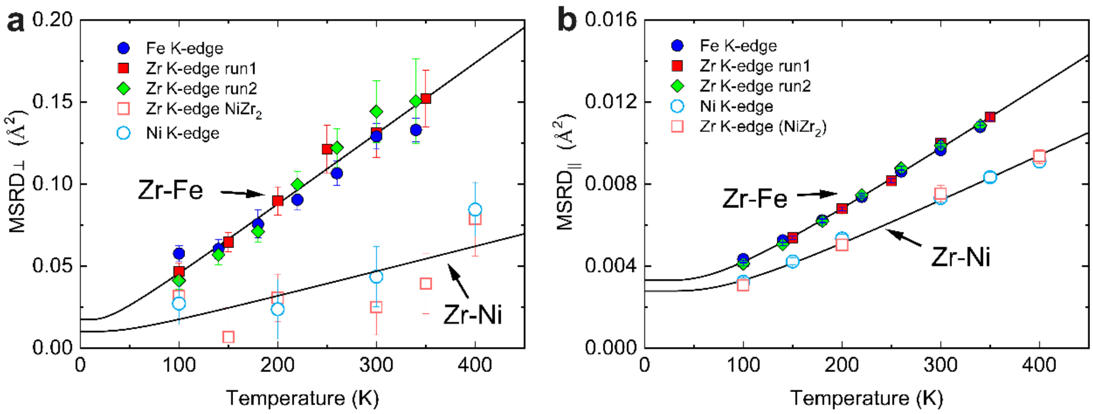

Fig. S17. The atomic mean-square relative displacements (MSRDs) extracted from EXAFS. (a) Perpendicular ($MSRD_{\perp}$) and (b) parallel ($MSRD_{\|}$) for Zr-M bond of MZr_2 (M = Fe, Ni). The bars represent calculated error values.

Table S2. The K-edge of Zr for $FeZr_2$.

Index	Path	Legs	Degeneracy	reff (Å)	Amplitude	Parameters
1	Zr-Fe	2	4	2.7290	100.00	r_1, σ_1^2, C_{31}
2	Zr-Zr	2	1	3.1091	42.306	r_2, σ_2^2
3	Zr-Zr	2	2	3.1095	21.145	r_2, σ_2^2
4	Zr-Zr	2	4	3.3181	71.912	r_2, σ_2^2
5	Zr-Zr	2	4	3.5505	60.294	r_2, σ_2^2

Table S3. The K-edge of Zr for $NiZr_2$.

Index	Path	Legs	Degeneracy	reff (Å)	Amplitude	Parameters
1	Zr-Ni	2	4	2.7611	100.00	r_1, σ_1^2, C_{31}
2	Zr-Zr	2	1	2.9889	25.531	r_2, σ_2^2
3	Zr-Zr	2	2	3.0790	47.517	r_2, σ_2^2
4	Zr-Zr	2	4	3.3767	75.326	r_2, σ_2^2
5	Zr-Zr	2	4	3.4322	72.195	r_2, σ_2^2

Table S4. The K-edge of Fe for $FeZr_2$.

Index	Path	Legs	Degeneracy	reff (Å)	Amplitude	Parameters
1	Fe-Zr	2	8	2.7290	100.00	r_1, σ_1^2, C_{31}
2	Fe-Fe	2	2	2.7877	18.961	r_2, σ_2^2 or neglected

Table S5. The K-edge of Ni for $NiZr_2$.

Index	Path	Legs	Degeneracy	reff (Å)	Amplitude	Parameters
1	Ni-Ni	2	2	2.6335	100.00	r_2, σ_2^2 or neglected
2	Ni-Zr	2	8	2.7611	495.947	r_1, σ_1^2, C_{31}

Table S6. Einstein frequency, bond effective force constants and anisotropy for the Zr-M bonds.[5]

	$FeZr_2$	$NiZr_2$
Zr-M bond stretching	$\upsilon_\parallel = 4.41 \pm 0.02$ THz<br>$\kappa_\parallel = 2.76 \pm 0.03$ eV/Å	$\upsilon_\parallel = 5.08 \pm 0.07$ THz<br>$\kappa_\parallel = 3.78 \pm 0.10$ eV/Å
Zr-M bond bending	$\upsilon_\perp = 1.67 \pm 0.04$ THz<br>$\kappa_\perp = 0.40 \pm 0.02$ eV/Å	$\upsilon_\perp = 2.77 \pm 0.44$ THz<br>$\kappa_\perp = 1.12 \pm 0.35$ eV/Å
Anisotropy $\gamma = 2\kappa_\parallel/\kappa_\perp$	13.9 ± 0.2	6.7 ± 0.9

5. First-principles calculations

5.1 Charge distribution of $FeZr_2$

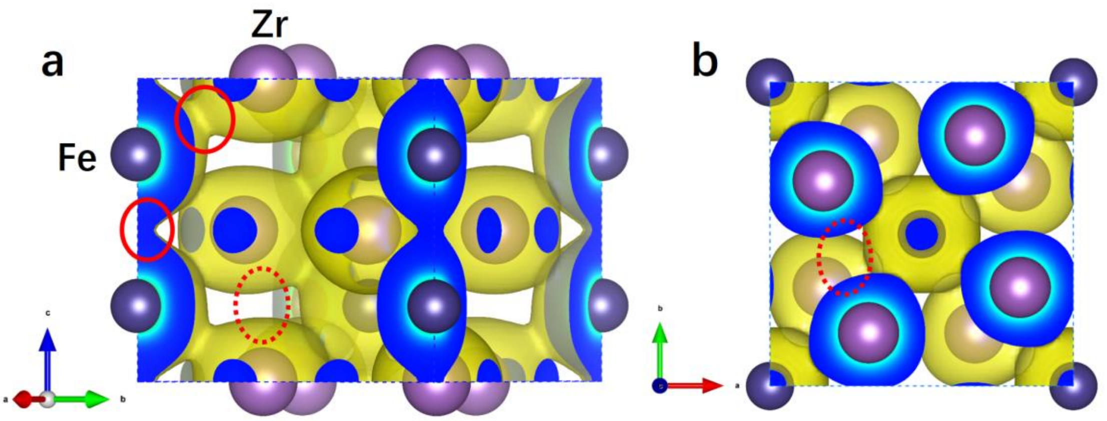

Fig. S18. Charge distribution. (a~b), Electron density distributions of $FeZr_2$ with a different perspective of observation and the same isosurfaces value at 0.0375 e/r_0^3 (r_0: Bohr radius). The red solid circles' mark represents the charged electron overlap section of Fe and Zr atom, and the red dashed circles mark the valence electron clouds without overlap for the interatomic distance > 3.2 Å between the nearest neighbor distance of Zr-Zr.

Generally, the bonding composition of intermetallic compounds is complex, especially for $CuAl_2$-type materials[2]. Different materials exhibit different bonding behaviors[6,7]. Here, the bonding of $FeZr_2$ between nearest-neighbor atoms can be determined by the isodensity charge density surface value, the interaction distances, and the -COHP (detailed discussion in Section 5.2). It can be found that the nearest-neighboring atoms pairs Zr-Fe and Fe-Fe charge clouds are the primary ones to overlap, which indicates the Zr-Fe and Fe-Fe bonds have strong interactions. In addition, it can be found that Zr-Zr with a distance less than 3.2 Å have relatively big -ICOHP values, indicating that less than 3.2 Å of Zr-Zr bonds also have strong interactions. Therefore, the bonding atom pairs are Zr-Fe, Fe-Fe, and Zr-Zr (interatomic distances less than 3.2 Å).

5.2 The energy bands, DOS, -COHP of MZr_2 systems

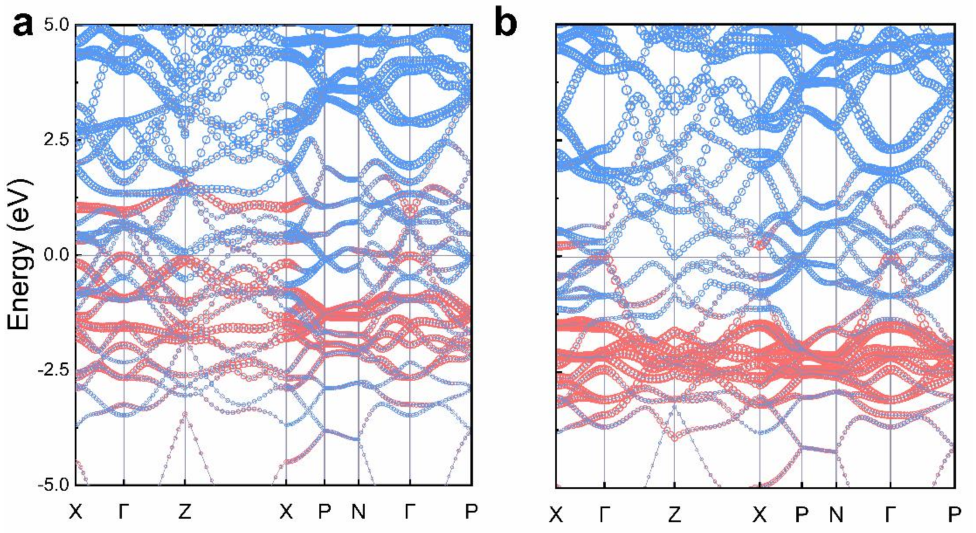

Fig. S19. The band structures of (a) $FeZr_2$ and (b) $NiZr_2$. The size of the circles corresponds to the magnitude of the band, the red color indicates Zr, and the blue color indicates M (M = Fe, Ni).

As shown in Fig. S19, we can find a lot of dispersive bands across the Fermi level in these three materials. The band structures show they are typical metallic systems. From the projected band structures, we can see it is mainly formed with Zr-character above the Fermi level and Fe-character below the Fermi level. Besides, we also see the flat band of M-character with red color moves down obviously from $FeZr_2$ to $NiZr_2$, due to the increasing number of 3*d*-electrons from Fe to Ni.

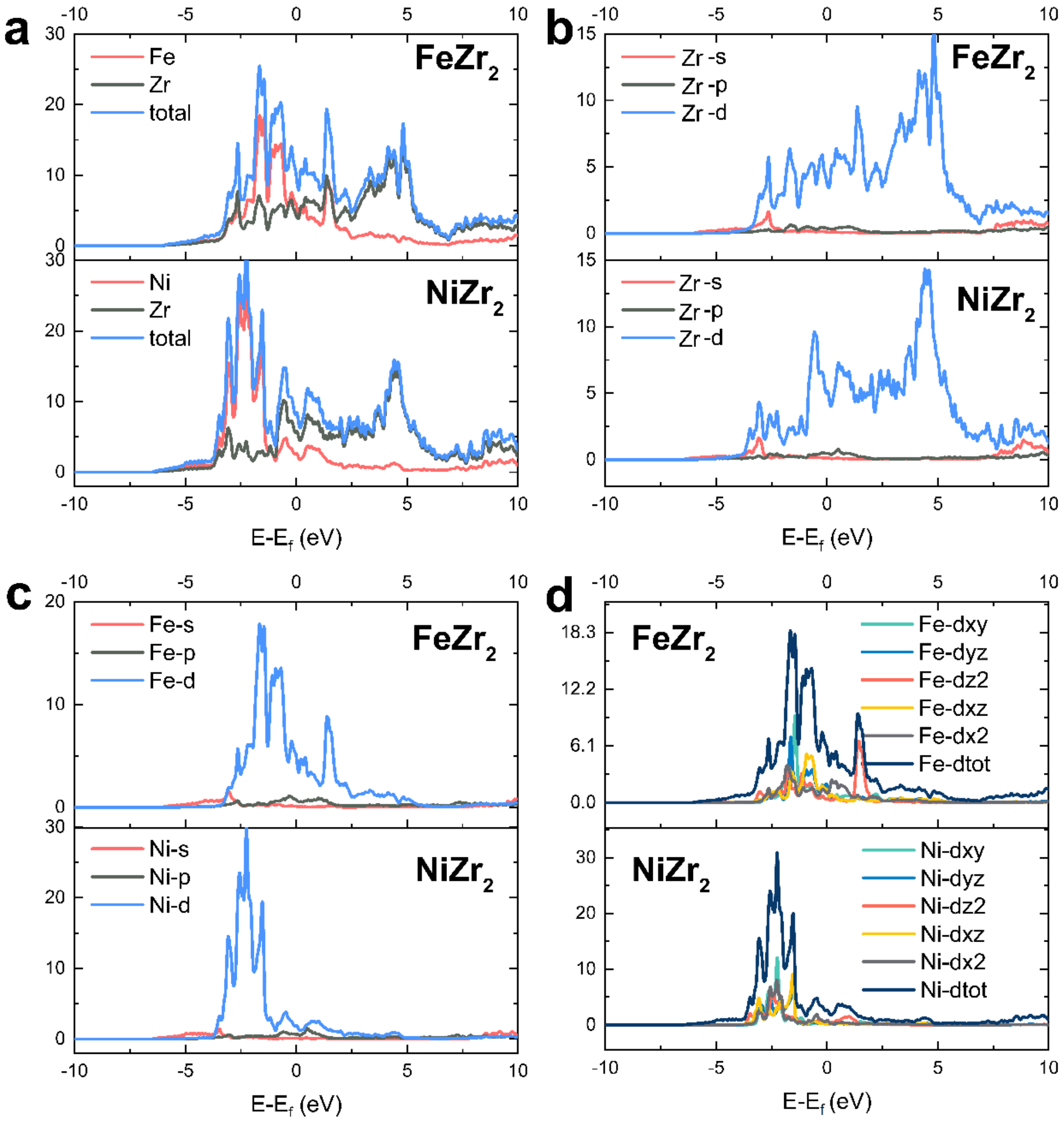

Fig. S20. The partial density of states of $FeZr_2$ and $NiZr_2$.

Figure S20(a) shows the total density of states (DOS) and partial density of states (pDOS) with different contributions of atoms in MZr_2. From the total DOS, we find there are considerable states near the Fermi level and the peak of DOS is relatively broad. These all show that MZr_2 are typical metallic material. Figure S20(b) gives the pDOS of M atoms. We find *d*-electrons of M atoms are near the Fermi level, while s and p electrons have a little contribution. The pDOS of M atoms is mainly below the Fermi level with occupation states. What's more, we can also see some *d*-electrons above the Fermi level. From Fe to Ni, the peak above the Fermi level becomes smaller and these peaks of pDOS move down continuously. Figure S20 (c) gives the pDOS of Zr-atoms. We find the *d*-electrons of Zr are mainly near the Fermi level and have a large

contribution. Compared with *d*-electrons of M, the change of *d*-electrons of Zr from Fe to Ni is not noticeable. Due to the itinerant *d*-electrons of M atoms, the five *d*-orbitals all cross the Fermi level, as shown in Fig. S20(d). However, in these five *d*-orbitals, we find that M-dz^2 is changing the largest. From Fe to Ni, we demonstrate the quick decrease of a peak above the Fermi level comes from dz^2-orbital.

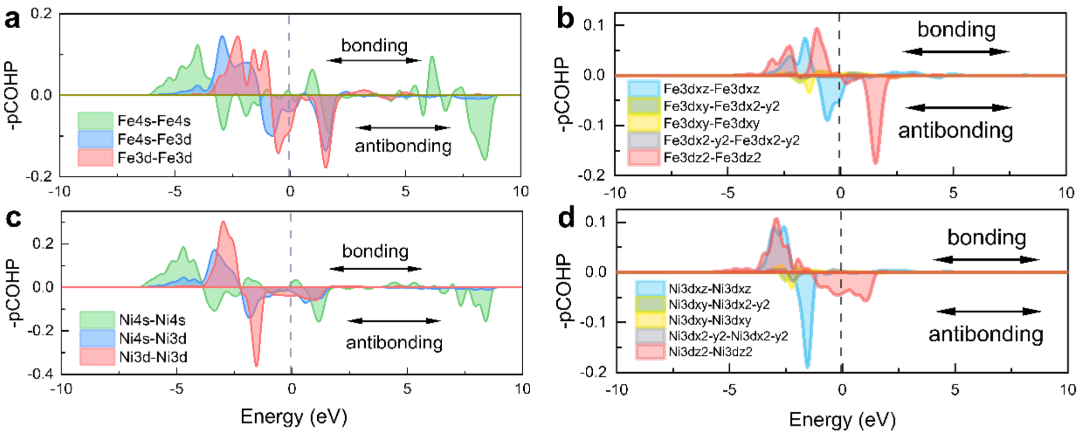

Fig. S21. The projected crystal orbital Hamilton population (-pCOHP) (eV/bond) data analysis for MZr_2 systems. The –pCOHP curves listed here are the main orbital-pair contributions for **(a)** Fe-Fe and **(c)** Ni-Ni. Detailed 3*d* orbital interactions in **(b)** Fe-Fe pair and **(d)** Ni-Ni pair. The Fermi levels, E_f, are indicated by the grey dotted lines.

As the number of M electrons increases, the 4s-4s, 4s-3d, and 3d-3d interactions of M-M all move toward the negative energy region (Fig. S21), which is consistent with DOS (Fig. S20). Meanwhile, it is found that the bonding and antibonding contributions of M3*d*-M3*d* interactions near the Fermi surface change most significantly from $FeZr_2$ to $NiZr_2$ (Fig. S21a,c). In addition, the interaction of M3dz^2-M3dz^2 is the largest variation near the Fermi surface, and the other interactions change slightly in all partial interactions of M3*d*-M3*d*. It means that the interactions of M3dz^2-M3dz^2 have a strong influence on M-M bonds.

Table S7. -ICOHP values (eV/bond•mol) of different MZr_2 components for different interatomic distances below 320 pm.

interaction	distance (Å)	multiple	-ICOHP
Fe-Fe	2.820(8)	4	-0.35
Zr-Fe	2.732(2)	32	-1.35
Zr-Zr	3.123(3)	8	-1.52
Zr-Zr	3.142(8)	4	-1.58

Table S8. The –IpCOHP/bond values (eV/bond•mol) and their percentage contributions of the respective interactions to the corresponding bonding –ICOHP. The –IpCOHP/bond values and the percentage contributions to the M-M bonds.

Interaction	-IpCOHP	%	-IpCOHP	%
	**Fe-Fe**		**Ni-Ni**	
M-4s – M-4s	-0.1269	36.43	-0.18035	55.22
M-3d-z^2 – M-3d-z^2	-0.10843	31.13	-0.05936	18.17
M-3d-z^2 – M-4s	-0.05675	16.29	-0.04837	14.81
M-4s – M-3d-z^2	-0.05583	16.03	-0.0483	14.79
M-3d-yz – M-3d-yz	-0.00152	0.44	0.00695	-2.13
M-3d-xz – M-3d-xz	-0.00149	0.43	0.00695	-2.13
M-3d-xy – M-3d-x^2-y^2	-0.00125	0.36	-0.00182	0.56
M-3d-x^2-y^2 – M-3d-xy	-0.00122	0.35	-0.00184	0.56
M-3d-xy – M-3d-xy	0.00211	-0.61	-0.00039	0.12
M-3d-x^2-y^2 – M-3d-x^2-y^2	0.00297	-0.85	-0.00008	0.02

References

1. Zhang, H. *et al.* Giant anisotropic magnetocaloric effect by coherent orientation of crystallographic texture and rare-earth ion moments in HoNiSi ploycrystal. *Acta Mater.* **193**, 210-220 (2020).
2. Havinga, E. & Damsma, H. Compounds and pseudo-binary alloys with the $CuAl_2$ (C16)-type structure III. Stability and competitive structures. *J. Less-Common Met.* **27**, 269-280 (1972).
3. Maurer, M., Friedt, J. & Sanchez, J. Local structure of amorphous and crystalline $FeZr_x$ (x = 2, 3) phases: Mossbauer spectroscopy and EXAFS investigations. *J. Phys. F: Met. Phys.* **15**, 1449 (1985).
4. Pauling, L. Atomic radii and interatomic distances in metals. *J. Am. Chem. Soc.* **69**, 542-553 (1947).
5. Sanson, A. EXAFS spectroscopy: a powerful tool for the study of local vibrational dynamics. *Microstructures* **1**, 2021004 (2021).
6. Armbrüster, M., Schnelle, W., Cardoso-Gil, R. & Grin, Y. Chemical bonding in compounds of the $CuAl_2$ family: $MnSn_2$, $FeSn_2$ and $CoSn_2$. *Chem. - Eur. J.* **16**, 10357-10365 (2010).
7. Armbrüster, M., Schnelle, W., Schwarz, U. & Grin, Y. Chemical bonding in $TiSb_2$ and VSb_2: A quantum chemical and experimental study. *Inorg. Chem.* **46**, 6319-6328 (2007).
